# A UPLC-DAD-Based Bio-Screening Assay for the Evaluation of the Angiotensin Converting Enzyme Inhibitory Potential of Plant Extracts and Compounds: Pyrroquinazoline Alkaloids from *Adhatoda vasica* as a Case Study

**DOI:** 10.3390/molecules26226971

**Published:** 2021-11-18

**Authors:** Syeda Tehreem, Saeedur Rahman, Muhammad Salman Bhatti, Reaz Uddin, Muhammad Noman Khan, Saba Tauseef, Hesham R. El-Seedi, Abdullatif Bin Muhsinah, Jalal Uddin, Syed Ghulam Musharraf

**Affiliations:** 1 H.E.J. Research Institute of Chemistry, International Center for Chemical and Biological Sciences, University of Karachi, Karachi 75270, Pakistan; tehreemwajid10@gmail.com (S.T.); saeedsohail4@gmail.com (S.R.); dr.salmanbhatti@gmail.com (M.S.B.); noman5937@gmail.com (M.N.K.); 2 Dr. Panjwani Center for Molecular Medicine and Drug Research, International Center for Chemical and Biological Sciences, University of Karachi, Karachi 75270, Pakistan; mriazuddin@iccs.edu (R.U.); sabatauseefrao@gmail.com (S.T.); 3 Pharmacognosy Group, Department of Pharmaceutical Biosciences, BMC, Uppsala University, SE-751 24 Uppsala, Sweden; 4 International Research Center for Food Nutrition and Safety, Jiangsu University, Zhenjiang 212013, China; 5 Department of Pharmacognosy, College of Pharmacy, King Khalid University, Abha 62529, Saudi Arabia; ajmohsnah@kku.edu.sa; 6 Department of Pharmaceutical Chemistry, College of Pharmacy, King Khalid University, Abha 62529, Saudi Arabia; jalaluddinamin@gmail.com; 7 T.C.M. Hospital of Southwest Medical University, Luzhou 646000, China

**Keywords:** angiotensin converting enzyme, hypertension, pyrroquinazoline alkaloids, vasicine, vasicinol, vasicinone

## Abstract

Angiotensin converting enzyme (ACE) plays a crucial role in regulating blood pressure in the human body. Identification of potential ACE inhibitors from medicinal plants supported the idea of repurposing these medicinal plants against hypertension. A method based on ultra-performance liquid chromatography (UPLC) coupled with a diode array detector (DAD) was used for the rapid screening of plant extracts and purified compounds to determine their ACE inhibitory activity. Hippuryl-histidiyl-leucine (HHL) was used as a substrate, which is converted into hippuric acid (HA) by the action of ACE. A calibration curve of the substrate HHL was developed with the linear regression 0.999. The limits of detection and quantification of this method were found to be 0.134 and 0.4061 mM, respectively. Different parameters of ACE inhibitory assay were optimized, including concentration, incubation time and temperature. The ACE inhibition potential of *Adhatoda vasica* (methanolic-aqueous extract) and its isolated pyrroquinazoline alkaloids, vasicinol (**1**), vasicine (**2**) and vasicinone (**3**) was evaluated. Compounds **1**–**3** were characterized by various spectroscopic techniques. The IC_50_ values of vasicinol (**1**), vasicine (**2**) and vasicinone (**3**) were found to be 6.45, 2.60 and 13.49 mM, respectively. Molecular docking studies of compounds **1**–**3** were also performed. Among these compounds, vasicinol (**1**) binds as effectively as captopril, a standard drug of ACE inhibition.

## 1. Introduction

Hypertension is a state in which the blood pressure is continuously elevated [1]. It is a major and common progressive disorder related to cardiovascular and renal diseases, stroke and diabetes [2]. Recent data have shown that one in six people in the world, or about one billion are afflicted by high blood pressure, and it is expected that this number is likely to increase to 1.5 billion by 2025 [3]. The World Health Organization said that hypertension is the most important cause of cardiovascular death [4]. The renin angiotensin aldosterone system (RAAS) or renin angiotensin system (RAS) plays a significant role in the regulation of blood pressure. ACE (EC 3.4.15.1) is an important physiological enzyme in the RAS which controls peripheral blood pressure and fluid balance in the body [5]. ACE is a di-peptidyl carboxypeptidase, first isolated from equine plasma, and it is present in biological fluids and many tissues [6]. It converts decapeptide angiotensin I to the potent vasoconstrictor octapeptide angiotensin II by the cleavage of the dipeptide histidyl-leucine [7].

Inhibition of ACE reduces the concentration of angiotensin II, which ultimately leads to a decrease in blood pressure [8]. Synthetic inhibitors such as lisinopril, captopril and enalapril are used as drugs for the treatment of hypertension; and many ACE inhibitory natural products and peptides have also been reported in the literatures which come from various plants and animal sources [5,6,9,10].

*Adhatoda vasica*, also known as *Justicia adhatoda*, is an important evergreen medicinal plant that belongs to the family Acanthaceae and is found in many areas of Pakistan and all over the world. It is widely used in various herbal formulations to treat the common cold and coughs, and it is very effective at treating respiratory complaints, such as asthma, muscle spasms, bronchitis, etc. [11]. Apart from the common cold and coughs, it is also used for other disorders, such as ear infections, urinary tract infections and rheumatic pain [12]. *A. vasica* possesses a large number of chemical constituents, but its most valuable phytochemicals are pyrroquinazoline alkaloids such as vasicine, vasicinone, vasicinol, vasicinolone, etc. These pyrroquinazoline alkaloids are responsible for its broad range of biological activities and increase its medicinal importance in Ayurvedic and Unani traditional medicine systems [13]. The ACE inhibitory potential of crude extracts of *A. vasica* has been reported earlier, but there is a lack of detailed study on the identification of key components responsible for this activity and on the determination of their ACE inhibitory potential [14].

**Scheme 1:**
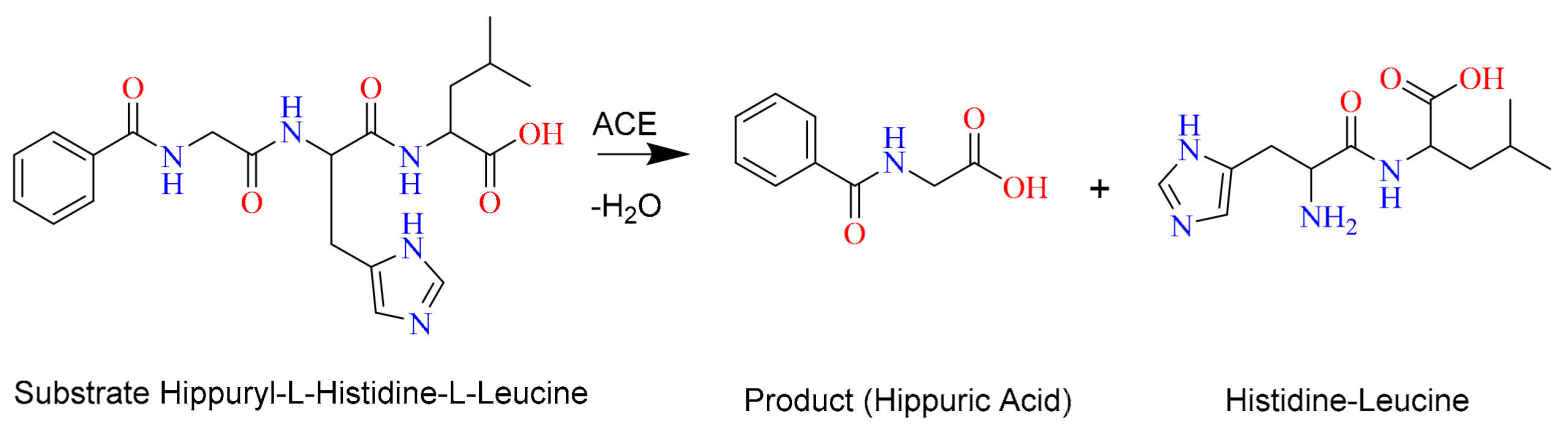
Hydrolysis of HHL into hippuric acid and histidine leucine by the action of ACE.

In this study, a UPLC-based enzymatic assay was used for the screening of plant extracts and natural products for the determination of ACE inhibitory activity. In this assay, HHL was used as a substrate which is converted into hippuric acid and histidine leucine by the action of ACE (Scheme 1). Three pyrroquinazoline alkaloids were isolated from *A. vasica* using a bioassay-guided approach and characterized by various spectroscopic techniques. Compounds 1–3 were studied for their ACE inhibitory potential. Moreover, molecular docking and ADME/Tox parameters of isolated compounds were also studied against ACE and compared with standard drugs.

## 2. Results and Discussion

### 2.1. Optimization of UPLC Conditions for the ACE Inhibitory Assay

**Figure 1:**
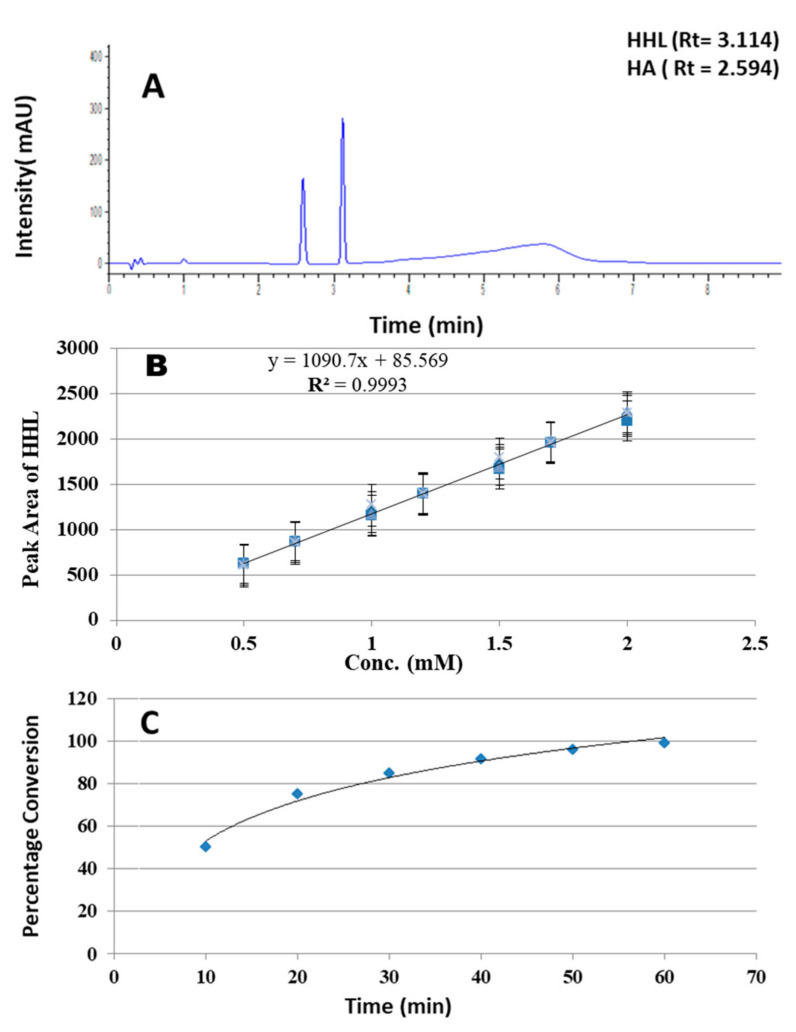
(**A**) UPLC-DAD chromatogram of HHL and HA. (**B**) Calibration curve of HHL. (**C**) ACE assay with different time intervals.

A UPLC and enzymatic assay optimization strategy from [15] was followed. Chromatographic columns with different column lengths (150, 100, 50 mm) were used for the optimization of chromatographic separation of the substrate and the product. The 150 mm and 100 mm columns required long run-times but provided better separation of substrate and product. However, the 50 mm column showed good separation within a small retention time (<4 min), so it was selected for our analysis. TFA and TEA were added in the mobile phase separately to check the effects on peak separation. Among these, TFA was found to be more suitable for the optimum separation of HHL and HA. The total run time was 9 min, which was good for the rapid screening of compounds as compared to the previously reported methods in which run time was 16 min or more [15,16]. Under optimized conditions, the retention time for the substrate HHL was found to be 3.1 min, and for HA it was found to be 2.6 min (Figure 1A). The baseline-separated peaks of substrate and product were observed within 3.5 min, which was good for the high-throughput analysis and increased selectivity.

The calibration range for HHL was selected as 0.5–2 mM, as the initial concentration of HHL in the enzymatic reaction was 1.5 mM at time 0 min. For the generation of calibration curve, seven different calibration levels were prepared, including 0.5, 0.7, 1.0, 1.2, 1.5, 1.7, and 2.0 mM. These calibration solutions showed good linear behavior in the calibration curve. The regression value was R² = 0.9993, and the regression equation was found to be y = 1090.7x + 85.569 (Figure 1B).

The limit of detection (LOD) and limit of quantification (LOQ) for the HHL were found to be 0.134 and 0.4061 mM, respectively. The method was found quite accurate and reproducible through intraday and inter-day analyses. For HHL determination, %RSD values were found to range from 0.08 to 2.20 and %error from 0.29 to 1.72 (Table 1).

### 2.2. Optimization of Angiotensin Converting Enzyme Assay

To optimize the enzymatic assay, different parameters such as temperature, enzyme concentration and incubation time were optimized. For temperature optimization, enzymatic reactions were performed at 25 and 37 °C separately, among which the enzyme showed better performance at 37 °C. At constant substrate concentration and temperature, three different concentrations of enzyme, 0.1, 0.4 and 1.5 U/mL, were also checked. At 0.1 U/mL ACE, the substrate was not consumed, and even after long incubation time of 50–60 min, very low conversion was observed. At 1.5 U/mL, substrate was consumed rapidly, which made it very difficult to optimize and observe enzymatic conversion. At 0.4 U/mL, the substrate was consumed partially, ~50–60%, in 20–30 min; therefore, the ACE concentration was selected as 0.4 U/mL for further experiments. To optimize the incubation time, an assay was performed, and 10 μL aliquots were taken at the intervals of 10, 20, 30, 40, 50 and 60 min. Each aliquot was treated and analyzed as before (Figure 1C). In 20 min of incubation, 60–70% of the substrate was converted into the product. This short incubation time is helpful for rapid and high-throughput analysis as compared to previously reported methods in which the incubation time was 30 min or more [6,15,17]. The adopted method has no time-consuming extraction steps as compared to many reported spectrophotometric assays, making it simpler and less time consuming [17].

In an enzymatic reaction, average initial reaction velocity can be calculated from the initial 20 min, because initially the enzyme will have a high velocity, and after some time, enzyme velocity decreases moderately. Average initial velocity can be calculated by using the initial points of percentage conversion vs. time graph (Figure 1C). ACE average initial velocity was calculated by using the following formula (Equation (1)).

(1) $$v^{0}=\frac{\mathrm{Cf}\;-\;\mathrm{Ci}}{tf\;-\;ti}$$

where v⁰ is the reaction initial velocity, Cf is the concentration of HHL at time t*f* and Ci is the initial concentration of HHL at time t*i*. The average reaction velocity for initial 20 min was found to be 0.056 mM/min for this ACE assay (Table S1, see Supplementary Material).

Rate constant can be calculated by plotting a graph between lnA (concentration of substrate) and time, in which the slope is the rate constant, by using the Equation (2); and the half-life of an enzymatic reaction is calculated by Equation (3).

(2) $${\mathrm{lnA}=\mathrm{lnA}}^{0}-\;\mathrm{Kt}$$

(3) $$t_{\frac{1\;}{2}}=\frac{0.693}{K}$$

The rate constant of this enzymatic reaction was found to be 0.0701 min^−1^, and the half-life was found to be 9.88 min.

Inhibition studies of ACE were performed by calculating the concentration of substrate. An increase in the concentration of substrate with comparison to the control would indicate inhibition, which was calculated as percent of maximal inhibition activity. Both lisinopril and captopril were checked for their inhibitory potential as a validation of the assay. For lisinopril, enzymatic reactions were performed at 100, 50 and 10 nM; and for captopril, 150, 100 and 50 nM concentrations were used. IC_50_ values of both drugs were calculated by plotting their percentages of inhibitory activity against concentration. The IC_50_ values for lisinopril and captopril were measured as 70.06 and 28.7 nM, respectively, which are shown in Figure S1 (see Supplementary Material). The ACE assay was found to be reproducible and accurate, with %RSD values ranging from 1.88 to 3.99 and %error from 2.20 to 4.04 (Table 2).

### 2.3. ACE Inhibitory Activity of Medicinal Plants and Purified Compounds

Total twenty-three plant extracts were screened for the investigation of ACE inhibitory activity, out of which ten extracts were found to be active. Plant extracts that showed inhibitory activity greater than 5% were considered as active, and plant extracts which showed activity less than 5% against ACE were considered as inactive. *Alpina galangal* and *Cissus quadrangularis* are traditionally used for cardiovascular diseases [18,19]. *Ziziphus vulgaris* was also previously reported as an ACE inhibitor [20]. Similarly, previous studies suggested that *Glycyrrhiza glabra* and *Viola odorata* have no ACE inhibitory activity, and these were also found inactive in this method [20]. ACE inhibitory activities of all the plants extracts are mentioned in Table S2 (see Supplementary Material).

Among the screened plants, *A. vasica* was also found active and was used for further experimentation. The methanolic-aqueous extract of *A. vasica* was further fractionated using n-hexane, dichloromethane, ethyl acetate and water. Each extract was subjected to a ACE inhibitory assay, but only the aqueous extract showed good ACE inhibitory activity with 13% ACE inhibition, and the rest were found inactive. Similarly, the 30% methanolic sub-fraction of aqueous extract showed the highest ACE inhibitory activity with 24% inhibitory activity among all the sub-fractions of aqueous extract, which led to the isolation of compounds **1**–**3**. Among all the compounds isolated from the 30% methanol, vasicine (**2**) had the highest ACE inhibitory activity and its IC_50_ was 2.60 mM. The IC_50_ values of vasicinol (**1**) and vasicinone (**3**) were found to be 6.45 and 13.49 mM, respectively.

### 2.4. Characterization of Pyrroquinazoline Alkaloids

Compounds **1**–**3** were successfully purified from the bioactive fraction (F1) by preparative recycling HPLC. The purity of compounds was checked by using HPLC-DAD, and they were found to be ≥95% pure (Figure S2, see Supplementary Material). The total ion chromatogram of the most active fraction F1 (with annotated peaks related to purified compounds) and LC-ESI-MS/MS based characterization of **1**–**3** are shown in Figure S3 (see Supplementary Material). The structures of compounds were confirmed by using FT-IR, ^1^H-NMR, ^13^C-NMR and LC-ESI-MS/MS. The FT-IR spectra of compounds **1**, **2** and **3** are shown in Figure S4 (see Supplementary Material). The structural characterization details for each compound are discussed below.

Compound **1** was obtained in crystalline solid form. FT-IR (KBr) ʋmax cm^−1^: 3337.8 (O-H stretch), 2957.3 (aliphatic C-H stretch), 3095.4 (aromatic C-H stretch) and 1689.9 (C=N stretch). Molecular formula (C_11_H_12_N_2_O_2_) was determined by using HR-ESI-MS, which showed a molecular ion peak [M + H]^+^ at *m/z* 205.0986. The HR-ESI-MS fragmentation data at 35 eV collision energy showed fragments at *m/z* (rel. abnd. %): *187.0878* (100), 169.0773 (13) and 159.0690 (15). This showed a base peak at *m/z* 187.0878 due to loss of H_2_O molecule (18 Da). ^1^H-NMR data of compound **1** showed characteristic signals at *δ* 6.99 (d, 1H, *J* = 8.8 Hz, H-5), 6.74 (dd, 1H, *J* = 8.8 and 2.4 Hz, H-6), 6.60 (d, IH, *J* = 2.8 Hz, H-8), 5.10 (t, 1H, *J* = 8.0 Hz, H-3) and 4.81–4.77 (each dd, 2H, *J* = 16.0 Hz, H-9). Chemical shifts values of the two methylenes proton at C-1 and C-2 positions were greatly affected by the chiral center present at C-3 and were multiplets at δ 3.74–3.60 (m, 2H) and 2.12–2.06 (m, 2H), respectively. The ^13^C-NMR (broadband and DEPT experiments) spectra showed 11 carbon signals, including 3 methylenes (CH_2_), 4 methines (CH) and 4 quaternary carbons. Proton and ^13^C-NMR spectra of compound **1** are shown in Figure S5 and S6 (see Supplementary Material) respectively.

Compound **2** was obtained as white needles. FT-IR (KBr) ʋmax cm^−1^: 32956.6 (O-H stretch), 2930.0 (aliphatic C-H stretch), 3054.8 (aromatic C-H stretch) and 1687.2 (C=N stretch). Molecular formula was determined to be C_11_H_12_N_2_O by using HR-ESI-MS, and its molecular ion peak [M + H] ^+^ was observed at *m/z* 189.1035. The HR-ESI-MS fragmentation showed ions at *m/z* (rel. abnd. %): 171.0928 (100), 169.0772 (57) and 167.0615 (9). This showed a base peak at *m/z* 171.0928 due to loss of a water molecule. Unlike compound **1**, the ^1^H-NMR spectra of compound **2** showed four aromatic protons signals at *δ* 7.33 (dt, 1H, *J* = 8.0 and 4.0 Hz, H-6), 7.25 (dt, 1H, *J* = 8.0 and 4.0 Hz, H-7), 7.18 (dd, IH, *J* = 4.0 Hz, H-5), 7.13 (dd, IH, *J* = 4.0 Hz, H-8). The remaining proton chemical shifts values of compound **2** were almost identical to those of compound **1**. The ^13^C-NMR spectrum also showed total of 11 carbon atoms, including three methylenes, five methines and three quaternary carbons. C-13 chemical shift values were very similar to those of compound **1**, except the aromatic carbon (CH) at C-7, which appeared at *δ* 128.34. ^1^H and ^13^C-NMR spectral data of compound **2** are shown in Figures S7 and S8 (see Supplementary Material), respectively. Compound **3** was isolated as **a** white amorphous solid. FT-IR ʋmax (KBr) cm^−1^: 3420.2 (O-H stretch), 2923.5 (aliphatic C-H stretch), 3002.8 (aromatic CH stretch), 1682.1 (C=O stretch) and 1470.4 (C-N stretch). C=O and C-N absorption peaks confirmed the presence of an amide group. Its molecular formula was found to be C_11_ H_10_N_2_O_2_ using HR-ESI-MS, and its molecular ion peak was observed at *m/z* 203.0821 [M + H]^+^. HR-ESI-MS/MS at *m/z* (rel. abnd. %): 185.0721 (100), 167.0618 (13), 158.0615 (2) and 157.0769 (1). Its base peak was observed at *m/z* 185.0717 (loss of one H_2_O molecule). The ^1^H and ^13^C-NMR spectral data of compound **3** showed typical peaks for an oxidized product (at C-9) of compound **2**. ^1^H-NMR showed four aromatic protons at *δ* 8.23 (dd, 1H, *J* = 8.0 and 1.1 Hz, H- 5), 7.83 (dt, 1H, *J* = 8.3 and 1.4 Hz, H-6), 7.74 (dd, 1H, *J* = 7.8 Hz, H-8) and 7.54 (dt, 1H, *J* = 8.1 and 1.1Hz, H-7). Protons at C-1 and C-2 appeared as multiplets and C-3 as a triplet, similarly to those of compounds **1** and **2**, but more downfield due to the carbonyl group (C=O) at C-9. ^13^C-NMR of compound **3** also showed 11 carbons, including four quaternary, two methylene and five methine carbons, similar to **1** and **2** but slightly downfield because of the carbonyl group. Proton and ^13^C-NMR spectra of compound **3** are shown in Figure S9 and S10 (see Supplementary Material), respectively.

**Figure 2:**
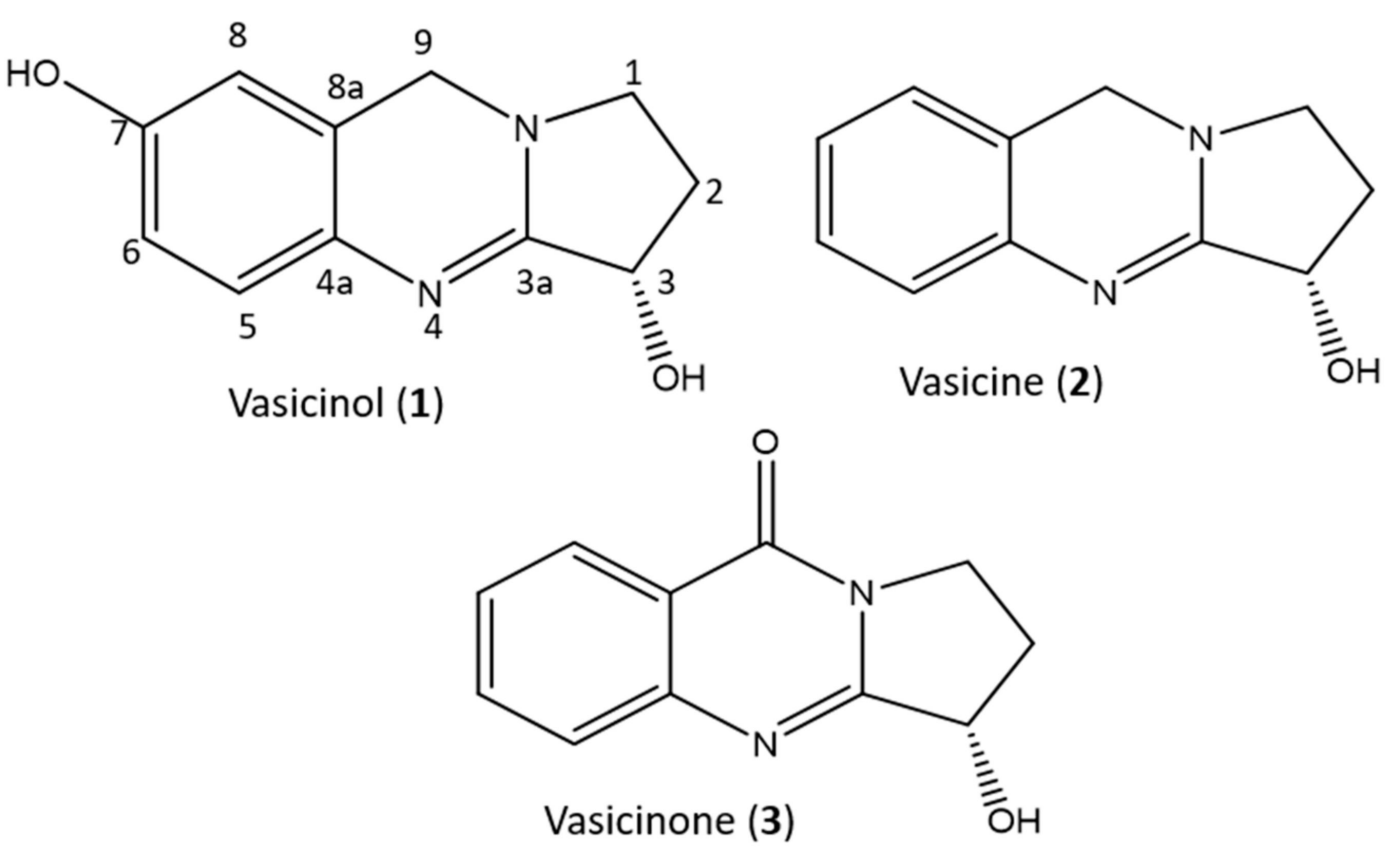
Chemical structures of purified compounds **1**–**3** from *Adhatoda vasica*.

The chemical structures of compounds **1**–**3** were elucidated as vasicinol (**1**), vasicine (**2**) and vasicinone (**3**); and their structures (Figure 2) were further confirmed by the comparison of observed ^1^H and ^13^C-NMR data (Table 1) with those in reported in literatures [21,22].

### 2.5. Molecular Docking

The active site for ACE was found through a literature survey, having amino acids Tyr520, Lys511, His513, His353, Ala354, Glu384, Tyr523, His387, His383, Asp377 and Glu162 [23]. The ligands and compounds were docked against the active site containing all essential residues.

The binding energies for lisinopril and captopril were calculated to be −8.48 and −6.97 kcal/mol, respectively (Figures S11 and S12, see Supplementary Material). Similar docking studies against ACE were also reported in the literature using lisinopril and captopril as standard inhibitors [23,24]. Our results showed that the binding energies of vasicine (**2**) and vasicinone (**3**) are comparatively lower than those of the known inhibitors, whereas vasicinol (**1**) showed significant binding energy. This suggests that ACE binds moderately with vasicine (**2**) and vasicinone (**3**), whereas vasicinol (**1**) binds effectively with ACE, as its binding energy is comparable to that of known inhibitor captopril. The binding interactions and binding free energies of compounds and inhibitors are mentioned in Table 3.

**Figure 3:**
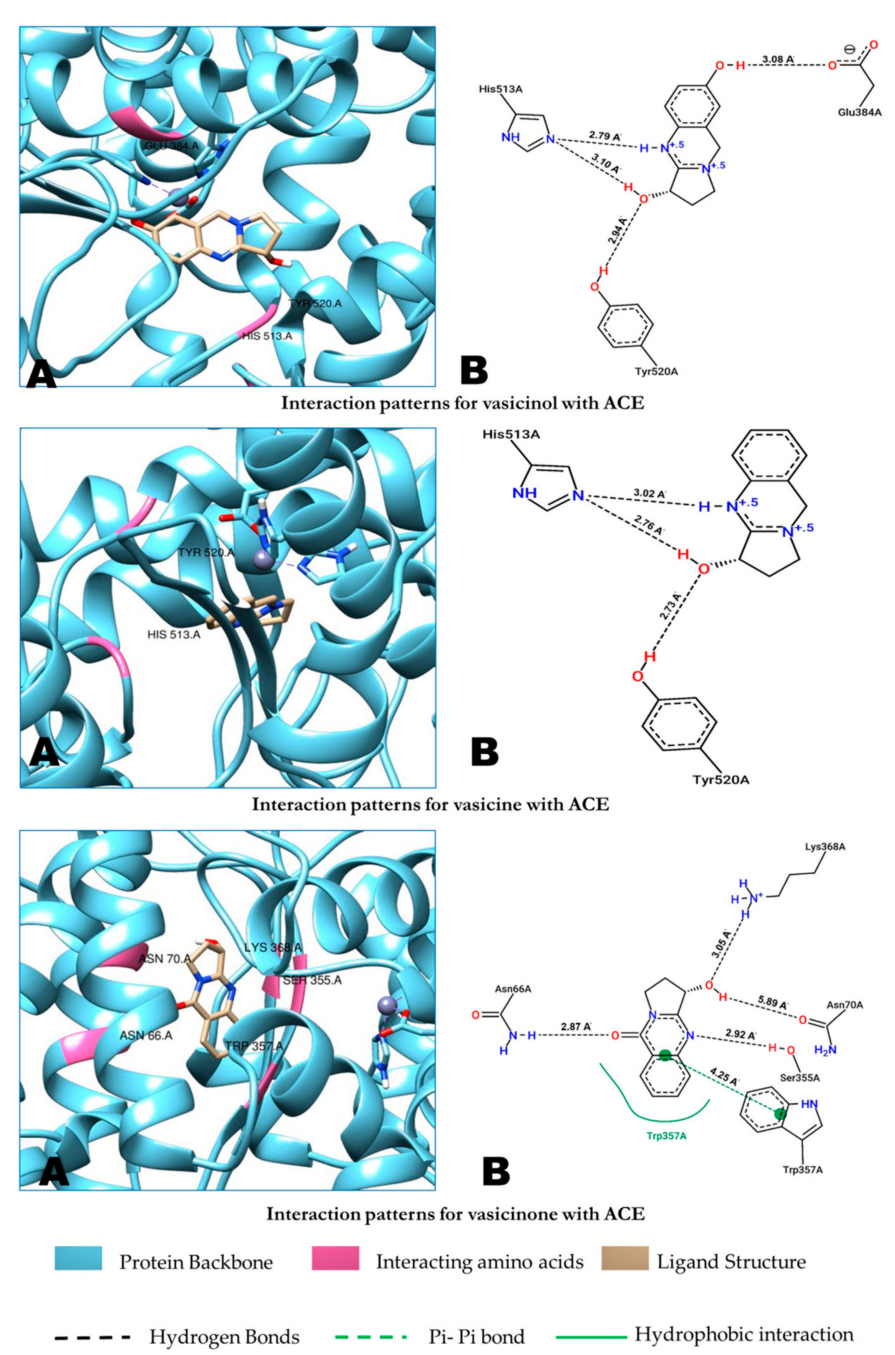
(**A**) Interactions of vasicinol (PubChem CID 442934) (**1**), vasicine (PubChem CID: 72610) (**2**) and vasicinone (PubChem CID: 442935) (**3**) with ACE (PDB. ID. 1O86), generated through Chimera. (**B**) 2D interactions of vasicinol (**1**), vasicine (**2**) and vasicinone (**3**) with ACE generated through PoseView.

The binding interaction diagrams for all the compounds are shown in Figure 3 Docking studies performed against ACE enzymes suggested that most of the active site residues, Tyr520, Lys511, His513, His353, Ala354, Glu384, Tyr523, His387, His383 and Glu162 [23], were involved in binding with known inhibitors; and vasicinol (**1**) and vasicine (**2**) mediated hydrogen bonds with His513 and Tyr520. Both compounds do not have any hydrophobic interactions with ACE enzymes. The compound vasicinone (**3**) showed interactions with different types of amino acid residues than the other compounds, and it also showed some hydrophobic interactions as well.

### 2.6. ADME/Tox Screening of Compounds

The compounds, which do not have any allergic or toxic properties and follow all the standard parameters of ADME/Tox properties, have strong drug-like molecule scores. The inhibitors and compounds **1**–**3**, when subjected to ADME/Tox analysis, showed that all the inhibitors and compounds follow the standard parameters and hence can act as potential drugs. The results of ADME/Tox analysis are shown in Table 4. The ADME/Tox analysis performed for the three compounds suggested that the compounds can act as potential drugs because they meet all the requirements of ADME/Tox analysis, and do not violate Lipinski’s rule of 5, have good solubility and have good oral bioavailability.

## 3. Materials and Methods

### 3.1. Chemicals and Reagents

ACE, N-hippuryl-l-histidyl-l-leucine and hippuric acid were purchased from Sigma-Aldrich Chemical Co. (St. Louis, MO, USA). Standard drugs lisinopril and captopril were obtained from Tokyo Chemical Industries Co., Ltd. (Tokyo, Japan); both were more than 98% pure. Boric acid was purchased from Sigma-Aldrich (Seelze, Germany). Acetonitrile was purchased from Merck, which was HPLC grade 99.9% pure. Deionized water was obtained from MiIIi-Q water assembly (Bedford, USA) and it was used in all the enzymatic reactions. Trifluoracetic acid (TFA) and Triethylamine (TEA) were purchased from Dae-jung Chemicals & Metals Co., Ltd. (Siheung-si, Korea). For the inhibition study, aqueous plant extracts (except for *Adhatoda vasica*, i.e., the methanolic-aqueous extract) were obtained from the Molecular bank, PCMD, University of Karachi.

### 3.2. UPLC-Based ACE Assay

The concentration of angiotensin converting enzyme was taken as 0.4 U/mL, and the concentration of substrate was 3 mM. Both ACE and substrate solutions were prepared in 10 mM of borate buffer. The borate buffer solution was freshly prepared at 10 mM, containing 300 mM NaCl; pH 8.3 was reached with 0.5 M NaOH at 37 °C. Standard inhibitor solutions of captopril (150, 100, 50 nM) and lisinopril (100, 50, 10, 1 nM) were prepared in water. For the screening of aqueous plant extracts, the solutions were prepared by dissolving dried plant extracts in water with the concentration of 4 mg/mL. The purified compounds were dissolved in DMSO to prepare solutions for screening against the enzyme. There was no significant difference observed in the enzymatic reaction in control experiments using DMSO and water.

All the samples were analyzed in UPLC system Agilent Technologies 1260 infinity California, United state coupled with DAD. Macherey-Nagel C−18 (3.0 × 50 mm, 1.8 μm) column was used for the chromatographic separation of substrate HHL and the product HA. The solvent system contained deionized water with 0.05% TFA as mobile phase A and acetonitrile with 0.05% TFA as mobile phase B, running at a constant flow rate of 0.8 mL/min, which was optimized prior to the experiments. The gradient system was started as 7% B, maintained for 0–0.5 min; then came 80% B for 3.5–3.8 min. Then it increased to 90% B for 4–4.6 min; and then decreased to 7% B for 5.1–9.0 min. The wavelength at which substrate and product were detected was 230 nm.

The linear calibration curve of HHL was prepared using series of solutions with concentrations as 0.5, 0.7, 1, 1.2, 1.5, 1.7 and 2 mM. All the calibration solutions were prepared from the stock solution of 3 mM HHL in borate buffer. Ten microliters of each sample were added to 10 µL of 1M HCl and analyzed thrice on UPLC with the injection volume of 5 μL. The calibration curve was plotted using the peak area and concentration of HHL.

### 3.3. Enzymatic Assay

Enzymatic reactions were performed in vials: each reaction mixture contained 25 μL 0.4 U/mL ACE, 50 μL 3 mM HHL substrate and 25 μL deionized water. Each reaction mixture had 100 μL of total volume. The enzymatic assays were performed in the incubator at 37 °C with constant shaking at 450 rpm. After incubation of 20 min, 10 µL of sample was taken and mixed with 10 µL of 1 M HCl to stop the reaction. Then, 5 μL of each sample was injected during each run into the UPLC system. For the analysis, triplicate runs were carried out to confirm the results.

The ACE activity was calculated by the quantification of substrate using the percentage conversion formula (Equation (4)), in which S is the initial concentration of the HHL and X is the concentration of HHL after a specific time.

(4) $$\%\;\mathrm{Conversion}=\frac{S\;-\;X}{S}\;\times \;100$$

Captopril and lisinopril are the standard drugs for ACE inhibition, and they were screened for the validation of the ACE inhibition assay. Once the parameters and reproducibility of ACE assay had been established, standard drugs were analyzed for their %inhibitory activity values, which were then used to determine the IC_50_ values.

Inhibitory activity was calculated from the given formula (Equation (5)):

(5) $$\;\mathrm{Inhibitory}\;\mathrm{Activity}=\left(1\;-\;\frac{\%\;C\;\mathrm{with}\;\mathrm{inhibitor}}{\%\;C\;\mathrm{without}\;\mathrm{inhibitor}}\right)\times \;100$$

In enzyme inhibition reactions, 25 μL of a standard drug solution (captopril and lisinopril; at different concentrations) was added instead of Milli Q water. Control reaction mixtures were also used. To measure the IC_50_ value of lisinopril, 100, 50, 10 and 1 nM solutions were used. For the IC_50_ of captopril, 150, 100, 50 nM solutions were used. The IC_50_ value is the concentration of a particular inhibitor at which it shows 50% inhibition of enzymatic activity. For the measurement of IC_50_ values_,_ 1, 5 and 10 mM solutions of vasicinol (**1**) and vasicine (**2**) were used; and 1, 10 and 20 mM solutions of vasicinone (**3**) were used.

### 3.4. Method Validation

The intraday and inter-day analyses for HHL detection and ACE inhibition assay were performed separately to determine precision and accuracy. For HHL, two quality control (QC) samples with 0.8 and 1.8 mM concentrations were used. For the validation of the bioassay, three solutions were analyzed for the inhibition of ACE, including lisinopril, captopril and a control, respectively. The intraday precision (as %RSD) was measured by analyzing each sample in triplicate within a day, whereas inter-day precision was determined by analyzing each sample on three successive days. The precision (as %RSD) was calculated from the standard deviation, and the mean value of observed concentration (Co) (Equation (6)) and the accuracy (% error) were calculated from the theoretical concentration (Ct) and the observed concentration (Co) (Equation (7)). Precision (% RSD) and accuracy (% error) were calculated by using following formulae:

(6) $$\mathrm{Precision}\;\left(\%\mathrm{RSD}\right)=\frac{\mathrm{Standard}\;\mathrm{deviation}}{\mathrm{Co}}\;\times \;100$$

(7) $$\;\mathrm{Accuracy}\;\left(\%\mathrm{Error}\right)=\frac{\mathrm{Ct}\;-\;\mathrm{Co}}{\mathrm{Ct}}\;\times \;100$$

The limit of detection (LOD) and the limit of quantification (LOQ) were calculated by (LOD = 3 σ/m, LOQ = 10 σ/m), in which σ is the standard deviation and m is the slope of calibration curve.

### 3.5. Extraction and Isolation of Compounds from Adhatoda vasica

The whole plant material of *A. vasica* was collected from district Bajaur, Khyber Pakhtunkhwa, Pakistan in 2019. The plant was identified as *A. vasica* by a plant expert, and a voucher specimen number (KUH-53882) was deposited in the Herbarium at Department of Botany, University of Karachi. 

Air-dried plant material of *A. vasica* (Approx. 2.5 kg) was ground, mixed to homogenous powder, immersed in 70% methanol/water (10 L), filtered after 5 days and then dried. The resulting dried residue (300 g) was suspended in minimum water (500 mL) and partitioned with n-hexane, dichloromethane, ethyl acetate (2L × 3, each) and finally, water. The organic and aqueous layers were dried under reduced pressure at room temperature. The dried aqueous layer (approx. 170 g) was adsorbed on reverse phase silica gel (RP C-18) and subjected to vacuum liquid chromatography. The fractions were eluted with 30%, 70% and 100% methanol in water, resulting in three fractions, F1, F2 and F3, respectively. Vacuum liquid chromatography (VLC) was used for fractionation by using silica gel (C-18-reverse phase, Merck). Fraction F1 (30% methanol) was obtained in good yield; and further, it was directly subjected to reversed phase preparative recycling HPLC using preparative recycling HPLC model (LC-908), fitted with JAI-ODS (L-80) column for purification of compounds **1**–**3**. HPLC-DAD profiles of purified compounds were achieved using HPLC (Agilent Technologies-1200 series) on a Macherey–Nagel gravity C-18 (3 × 100 mm, 1.8 µm) column with injection volume 5 μL, flow rate 0.5 mL/min and total run time 10 min including 1 min of equilibration time. The mobile phase comprised of 0.1% aqueous formic acid (eluent A) and 0.1% formic acid in methanol (as eluent B). A gradient solvent system starting from 10% B, gradually increasing up to 90% B in 5.50 min, staying at 90% for 1.50 min and returning back to 10% B for 1 min was used.

### 3.6. Characterization of Pyrroquinazoline Alkaloids

The infrared (IR) spectra of purified compounds **1**–**3** were recorded on an FT-IR machine (Shimadzu-8900, Japan) using the KBR disc method. ^1^H and ^13^C-NMR spectra were recorded on Bruker Avance-NMR instruments (^1^H-NMR 400 and 500MHz, ^13^C-NMR 150 and 75 MHz) using CD_3_OD as a solvent. Multiplicities of carbon-13 signals were defined by using DEPT-90° and 135° experiments. HR-ESI-MS spectra of compounds **1**–**3** were recorded on a mass spectrometer (ESI-QTOF, Bruker maXis II™, Bremen, Germany) coupled with reverse phase UPLC (Thermo ultimate-3000). ^1^H and ^13^C-NMR values for compounds **1**–**3** are mentioned in Table 5.

### 3.7. Molecular Docking Screening

The compounds vasicinol (**1**) (PubChem CID 442934), vasicine (**2**) (PubChem CID: 72610) and vasicinone (**3**) (PubChem CID: 442935) were retrieved from PubChem [25]. The target enzyme ACE [26] (PDB. ID. 1O86), and two known inhibitors lisinopril [26] (PDB. ID. LPR) and captopril [27] (PDB. ID. X8Z), were retrieved from Protein Data Bank. The energy minimization, along with conversion of compounds (**1**–**3**) into 3D PDB format, were done using FROG2 tool [28]. The protein ACE was prepared for docking using UCSF Chimera [29] involving removal of water molecules and ligands from the parent protein chain.

The two known inhibitors, lisinopril and captopril, were used for validation of molecular docking onto the target protein ACE using Autodock 4.2 [30]. The grid box was set to 50 ∗ 50 ∗ 50 Å along x, y and z axes and centered on 40.553, 32.798 and 47.286 in which the grid box was centered on ligand lisinopril. The dockings were performed using standard set parameters and the Lamarckian genetic algorithm (LGA) for 250 runs. The results were evaluated using root mean square deviation (RMSD) values, also known as the docking scores or binding free energies of docked compounds. The ligand–protein interaction was visualized using Chimera, and the 2D interaction between protein and ligand was visualized using PoseView [31]. The same grid box and procedure was used for docking of captopril and three energy minimized compounds against the target protein ACE, while keeping all other parameters same. The docked ligands with the best RMSD values were analyzed through Chimera and PoseView.

### 3.8. ADME/Tox Screening of Compounds

The two known inhibitors and three compounds (**1**–**3**) were screened for their ADMET (i.e., absorption, distribution metabolism, excretion and toxicity) through FAF*Drugs*4 using Mobyle@rpbs server [32]. The 3D sdf (structure data format) forms of inhibitors and compounds were retrieved from PubChem (lisinopril PubChem CID: 5362119, captopril PubChem CID: 44093, vasicinol PubChem CID: 442934, vasicine PubChem CID: 72610, vasicinone PubChem CID: 442935). The compounds were submitted in 3D sdf format to calculate numbers of drug-like parameters. The drug-like parameters must have values in between ranges given below:

Molecular weight: 100.0–600.0, hydrogen bond donors: ≤7, hydrogen bond acceptors: ≤12, rotatable bonds: ≤11, rigid bonds: ≤30, ring number: ≤6, ring size: ≤18, number of carbons: 3–35, heteroatoms: 1–15, ratio carbon/hetero: 0.1–1.1, charge number: ≤4, total charge: −4.0–4.0, logP: −3.0–6.0, polar surface area: ≤180 [33,34,35,36,37].

## 4. Conclusions

In this study, a UPLC-based enzymatic assay was optimized and used for the screening of potential inhibitors of angiotensin converting enzyme. Some common medicinal plants were screened for their ACE inhibitory activity among which the methanolic-aqueous extract of *A. vasica* has shown significant activity. Bioassay-guided approach was used to identify the bioactive compounds from active *A. vasica* fraction. This leads to the identification of three pyrroquinazoline alkaloids **1**–**3**. Among these compounds, vasicine (**2**) has shown the highest ACE inhibitory potential. Although, in comparison with standard drugs, the activities of compounds are not significant, the results will encourage future studies by UPLC-based enzymatic assay to explore the therapeutic use of medicinal plants. The purified alkaloids were also screened through molecular docking studies against ACE enzyme for their inhibitory potential. Vasicine (**2**) and vasicinone (**3**) have shown moderate binding energies among them while vasicinol (**1**) was found to bind as effectively as captopril which is the known inhibitor drug. Furthermore, twenty-two other plants have also been screened for their ACE inhibitory potential. Many of these plants have shown good ACE-inhibitory activities. Due to substantial ACE-inhibitory activity, natural source, easy availability, and less side effects than a synthetic drug, the medicinal plants can be effectively used against hypertension after necessary in vivo study. The developed strategy can also be applied for the discovery of lead molecules from other plant sources and high-throughput searching of new antihypertensive compounds, plants or food material.

## Figures and Tables

**Table 1:** Intraday and inter-day analysis to determine the precision and accuracy of HHL.

S.No.	HHL Conc. (mM)	Intraday			Inter-Day		
		Mean Conc. (mM)	RSD %	%Error	Mean Conc. (mM)	RSD %	%Error
1.	0.8	0.8023	1.0850	0.2929	0.8137	2.2019	1.7230
2.	1.8	1.8232	0.0841	1.2921	1.8160	0.6852	0.8932

**Table 2:** Intraday and inter-day analysis to determine the precision and accuracy of ACE assay.

S.No.	ACE Assay	Intraday			Inter-Day		
		ACE %Conversion	RSD %	%Error	ACE %Conversion	RSD %	%Error
1.	Control	70.0940	1.8826	3.0795	69.5008	2.7430	2.2071
2.	Captopril (100 nM)	28.0923	2.6146	4.0457	27.8100	3.0499	3.0000
3.	Lisinopril (100 nM)	21.8837	3.9631	4.0187	21.8887	3.9927	3.9969

**Table 3:** Binding free energies of inhibitors/compounds, along with interacting residues of ACE protein.

Inhibitors/Compounds	Binding Free Energy Kcal/mol	Types of Interactions Along with Interacting Amino Acids of ACE
Lisinopril	−8.48 kcal/mol	Hydrogen Bonds Glu162, Gln281, His353, Lys511, His513, Tyr520 and Tyr523 Hydrophobic Interactions His 353, Ala354, Ala356, Val380 and His387
Captopril	−6.97 kcal/mol	Hydrogen Bonds Gln 281, His353, Glu384, Lys511, Tyr520, Tyr523 and His513 Hydrophobic Interactions His383 and Tyr 523
Vasicine	−5.89 kcal/mol	Hydrogen Bonds His513 and Tyr520
Vasicinol	−6.40 kcal/mol	Hydrogen Bonds Glu384, His513, and Tyr520
Vasicinone	−5.79 kcal/mol	Hydrogen Bonds Asn66, Asn70, Ser355, Trp357 and Lys368 Hydrophobic Interactions Trp357

**Table 4:** Analysis of compounds using FAF*Drugs*4 for ADME/Tox properties.

Ligand	Parameters Standards	Lisinopril	Captopril	Vasicinol	Vasicine	Vasicinone
PubChem ID	–	5362119	44093	442934	72610	442935
Mol. Weight	100.0–600.0	405.49	217.29	204.23	188.23	202.21
LogP	−3.0–6.0	−1.22	0.34	0.09	0.44	0.52
HBA	≤12	8	4	4	3	4
HBD	≤7	5	1	2	1	1
Tpsa	≤11	144.82	99.24	57.67	37.44	55.12
RotatableB	≤11	12	3	0	0	0
RigidB	≤30	15	8	15	15	16
Rings	≤6	2	1	1	1	1
Max Size Ring	≤18	6	5	13	13	13
Carbon Atoms	3–35	21	9	11	11	11
Hetero Atoms	1–15	8	5	4	3	4
Ratio H/C	0.1–1.1	0.38	0.56	0.36	0.27	0.36
NumCharges	≤4	4	1	1	1	0
Total Charge	−8	0	−1			
Lipinski Violation	–		0	0	0	
Solubility(mg/L)		170317.77	65224.97	54156.75	43933.32	36306.82
Solubility Forecast Index		Good Solubility	Good Solubility	Good Solubility	Good Solubility	Good Solubility
Oral Bioavailability (VEBER)		Low	Good			
Oral Bioavailability (EGAN)						
StereoCenters		3	2	1		
Result		Accepted				

**Table 5:** **^1^**H and ^13^C-NMR spectral data for compounds **1**–**3** in CD_3_OD.

Position	Compound 1		Compound 2		Compound 3	
	*δ*_H_ (mult., *J* in Hz)	*δ*_C13_	*δ*_H_ (mult., *J* in Hz)	*δ*_C13_	*δ*_H_ (mult., *J* in Hz)	*δ*_C13_
1	3.75–3.61 (m)	53.02	3.33–3.45 (m)	47.52	4.29–4.02 (m)	44.58
2	2.12–2.06 (m)	31.76	2.41–2.18 (m)	30.78	2.66–2.18 (m)	31.08
3	5.11 (t, 8.0)	73.46	4.66 (t, 6.4)	72.54	5.12 (t, 7.8)	73.26
3a	---	163.77	---	164.76	---	161.84
4a		126.22		132.43		150.47
5	6.98 (d, 8.8)	121.38	7.18 (d, 8.1)	130.47	8.24 (dd, 8.2, 1.2)	135.67
6	6.75 (dd, 8.8, 2.4)	118.54	7.33 (dt, 8.0, 4.0)	128.35	7.84 (dt, 8.3, 1.4)	128.00
7	---	157.75	7.25 (dt, 8.0, 4.0)	128.26	7.55 (dt, 8.1, 1.2)	127.16
8	6.62 (d, 2.8)	116.23	7.13 (d, 8.0)	118.42	7.73 (d, 7.8)	128.01
8a	---	121.18	---	118.24	---	121.91
9	4.81–4.77, 2H (d, 16.0)	48.89	5.08 (d, 15)	51.95		162.59

## Data Availability

The supporting data is available as supplementary material.

## Supplementary Materials

The following are available online. Figure S1. ACE inhibition assay by (A) Captopril and (B) Lisinopril. Figure S2. HPLC-DAD profiles of compounds **1**, **2** and **3** purified from fraction (F1) using preparative recycling HPLC. Figure S3. Total ion chromatogram (TIC) of the most active fraction with annotated peaks related to purified compounds (A) and LC-ESI-MS/MS based characterization of compounds **1**–**3** shown in (B, C and D), respectively. Figure S4. FTIR spectrum of Compound **1**, **2** and **3**. Figures S5–S10. Proton and C-13 NMR spectrum of compound **1**, **2** and **3**. Figure S11. (A) 2D Structure of lisinopril, (B) 3D structure of lisinopril, (C) 3D interaction of lisinopril with ACE generated through Chimera, and (D) 2D interactions of lisinopril with ACE generated through PoseView. Figure S12. (A) 2D Structure of captopril, (B) 3D structure of captopril (C) 3D interaction of captopril with ACE generated through Chimera, and (D) 2D interactions of captopril with ACE generated through PoseView. Table S1. Reaction initial velocity of ACE in enzymatic assay. Table S2. ACE inhibitory activity of plant extracts.
