# Supplementary material for: A UPLC-DAD-Based Bio-Screening Assay for the Evaluation of the Angiotensin Converting Enzyme Inhibitory Potential of Plant Extracts and Compounds: Pyrroquinazoline Alkaloids from Adhatoda vasica as a Case Study
Source: Molecules. 2021 Nov 18;26(22):6971. doi: 10.3390/molecules26226971 (PMC8617709; doi:10.3390/molecules26226971)
Supplement: Supplementary file 1 [file molecules-26-06971-s001.zip › molecules-1423328-supplementary.pdf]

Supplementary material

Supplementary Figure Legends

Figure S1: ACE inhibition assay by (A) Captopril and (B) Lisinopril.

Figure S2: HPLC-DAD profiles of compounds **1, 2** and **3** purified from fraction (F1) using preparative recycling HPLC.

Figure S3: Total ion chromatogram (TIC) of the most active fraction with annotated peaks related to purified compounds (**A**) and LC-ESI-MS/MS based characterization of compounds **1-3** shown in (**B, C** and **D**), respectively.

Figure S4 : FTIR spectrum of Compound **1**,**2** and **3.**

Figure S5: Proton NMR spectrum of compound **1**.

Figure S6: C-13 NMR spectrum of compound **1.**

Figure S7: Proton NMR spectrum of compound **2**.

Figure S8: C-13 NMR spectrum of compound **2.**

Figure S9: Proton NMR spectrum of compound **3**.

Figure S10: C-13 NMR spectrum of compound **3**.

Figure S11: (A) 2D Structure of lisinopril, (B) 3D structure of lisinopril, (C) 3D interaction of lisinopril with ACE generated through Chimera, and (D) 2D interactions of lisinopril with ACE generated through PoseView.

Figure S12: (A) 2D Structure of captopril, (B) 3D structure of captopril (C) 3D interaction of captopril with ACE generated through Chimera, and (D) 2D interactions of captopril with ACE generated through PoseView.

Supplementary Table Legends

Table S1: Reaction initial velocity of ACE in enzymatic assay.

Table S1: ACE inhibitory activity of plant extracts.

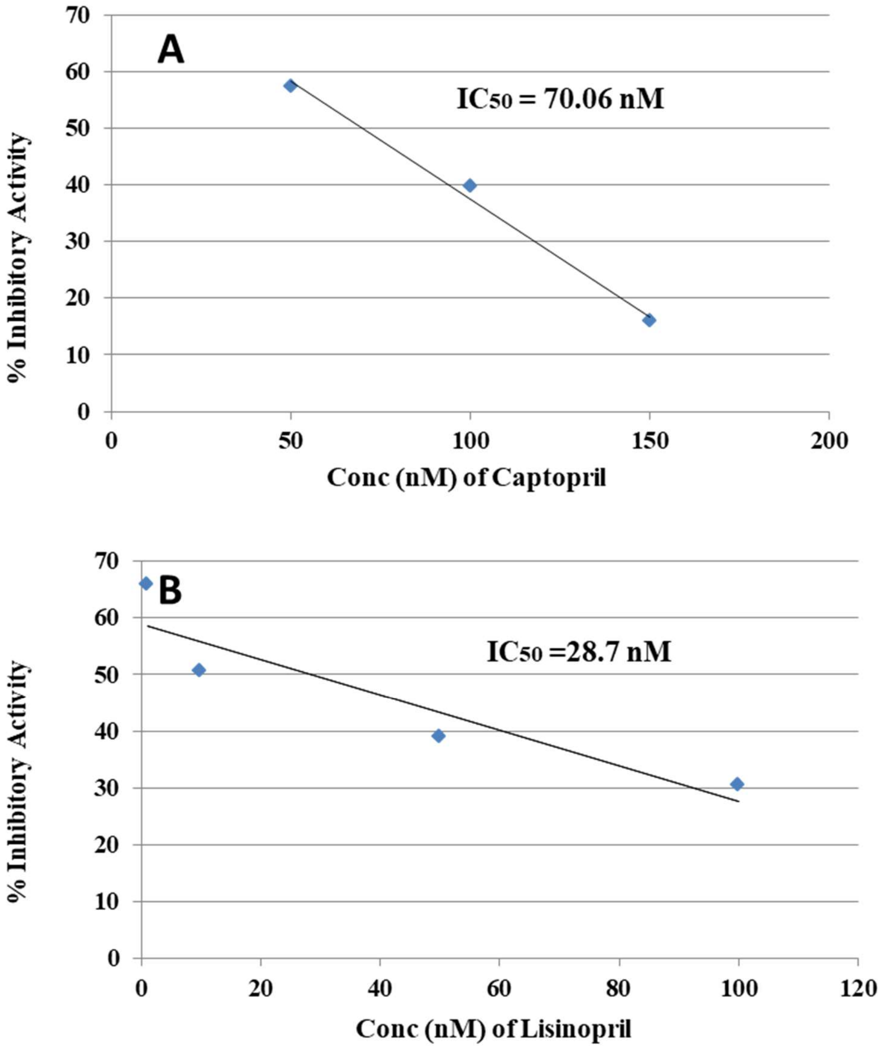

Figure S1: ACE inhibition assay by (A) Captopril and (B) Lisinopril.

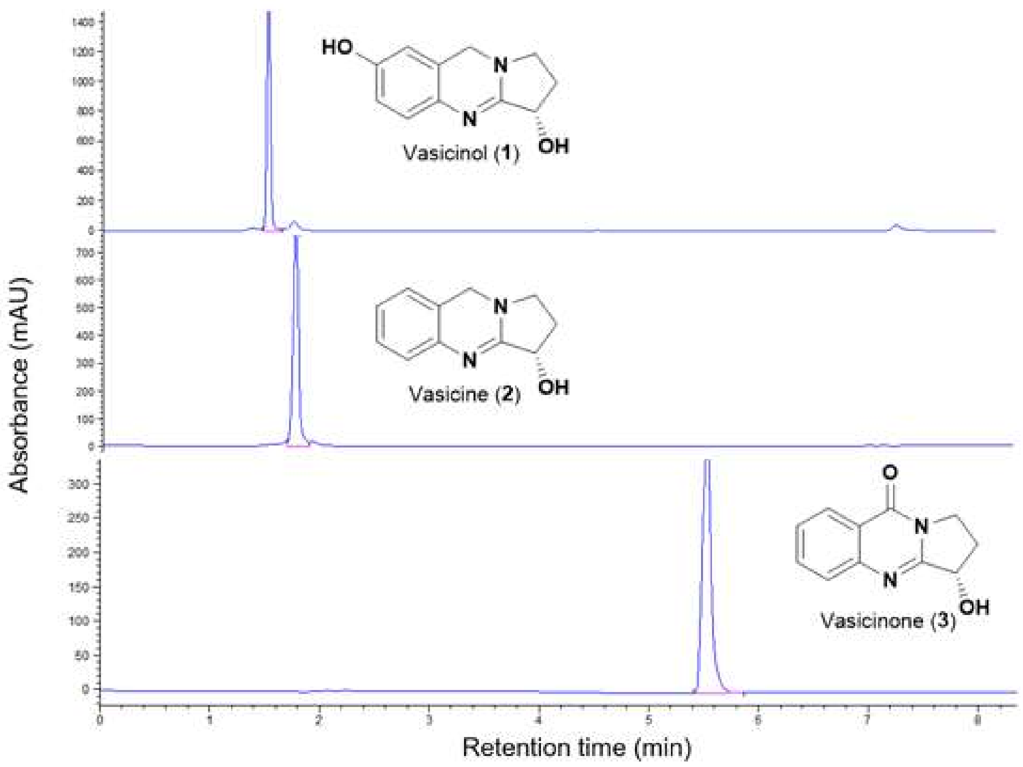

Figure S2: HPLC-DAD profiles of compounds **1, 2** and **3** purified from fraction (F1) using preparative recycling HPLC.

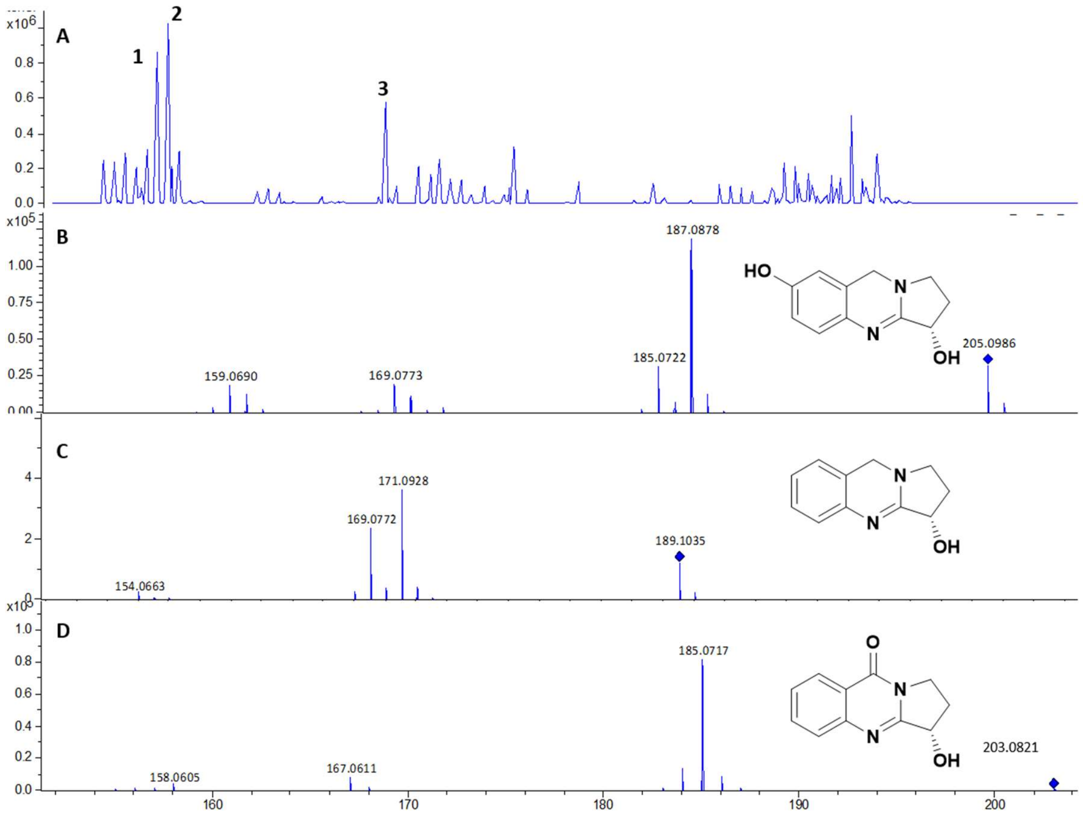

Figure S3: Total ion chromatogram (TIC) of the most active fraction with annotated peaks related to purified compounds (**A**) and LC-ESI-MS/MS based characterization of compounds **1-3** shown in (**B, C** and **D**), respectively.

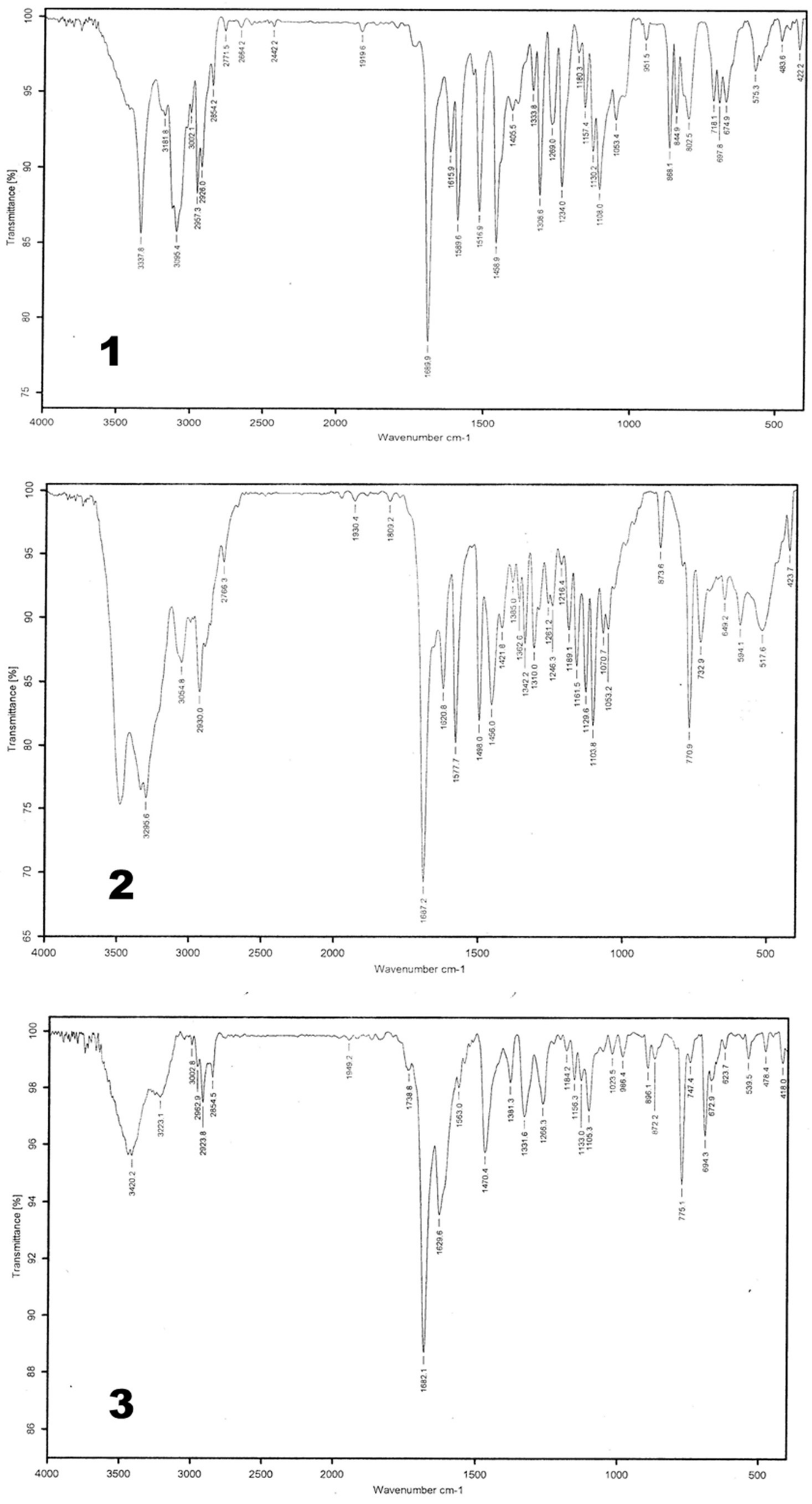

Figure S4 : FT-IR spectrum of Compound **1**,**2** and **3.**

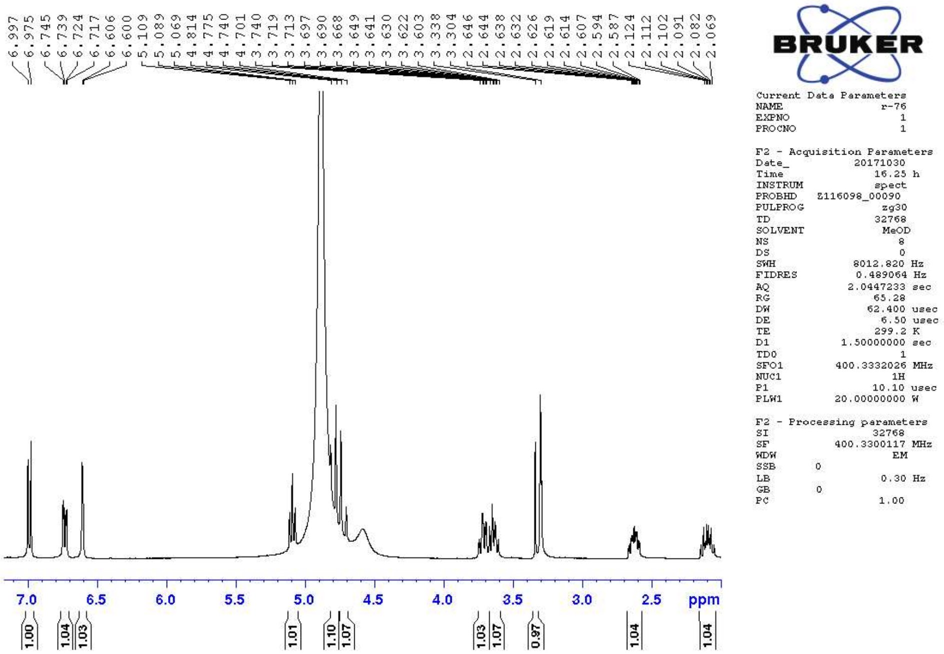

Figure S5: Proton NMR spectrum of compound **1**.

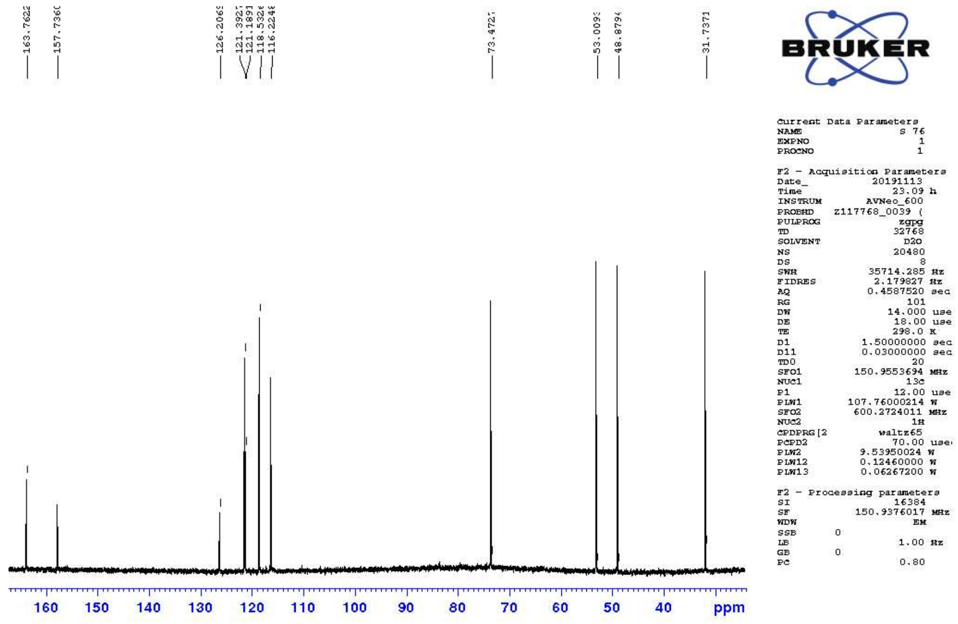

Figure S6: C-13 NMR spectrum of compound **1.**

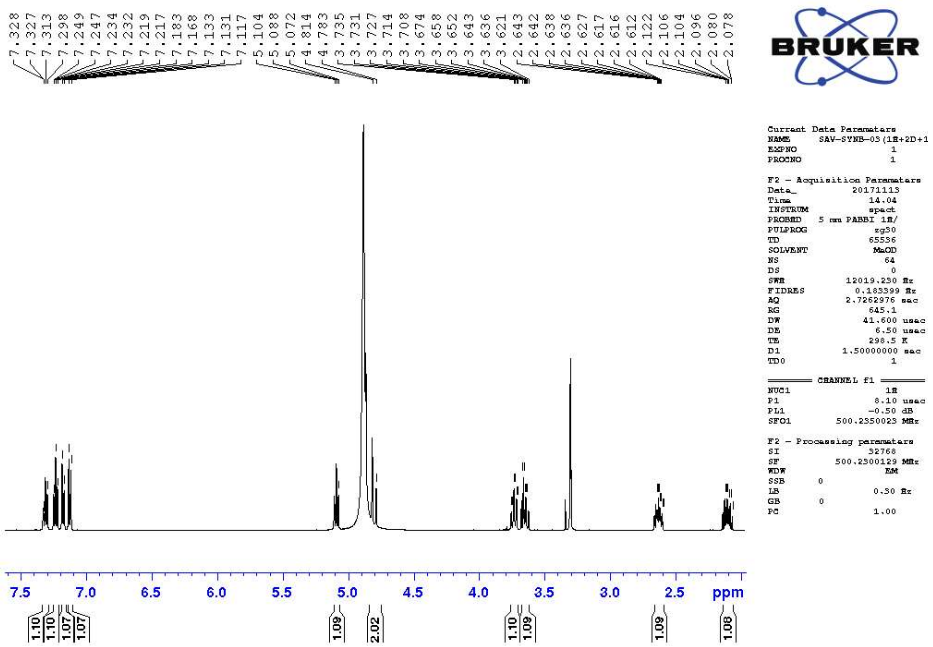

Figure S7: Proton NMR spectrum of compound **2**.

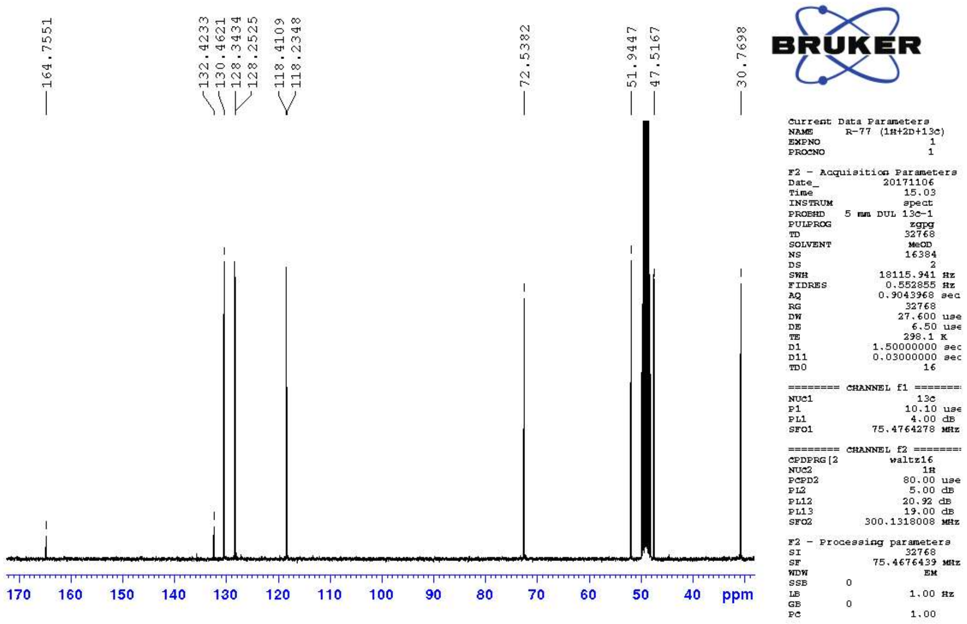

Figure S8: C-13 NMR spectrum of compound **2.**

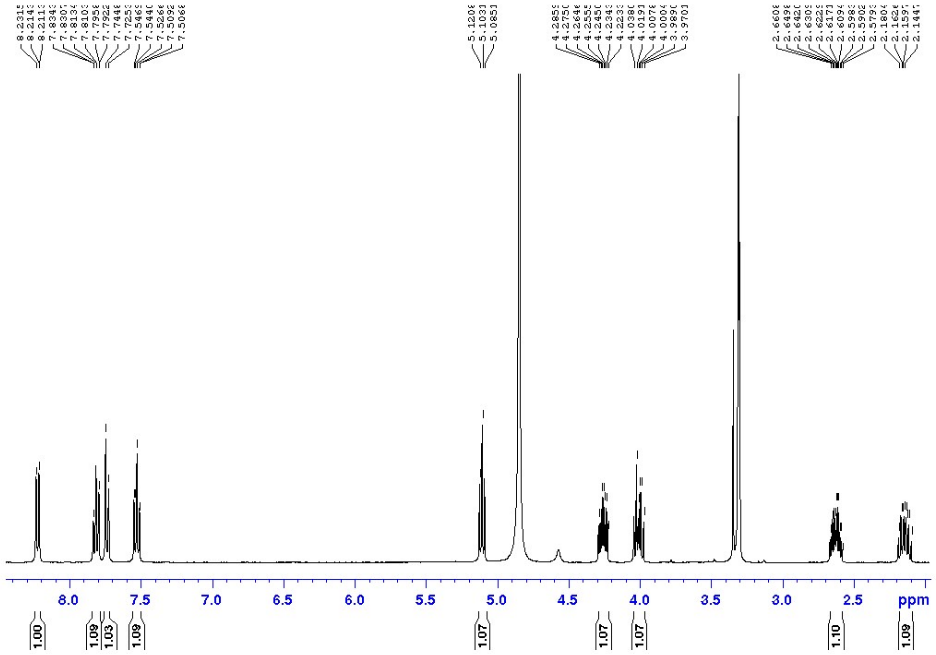

Figure S9: Proton NMR spectrum of compound **3**.

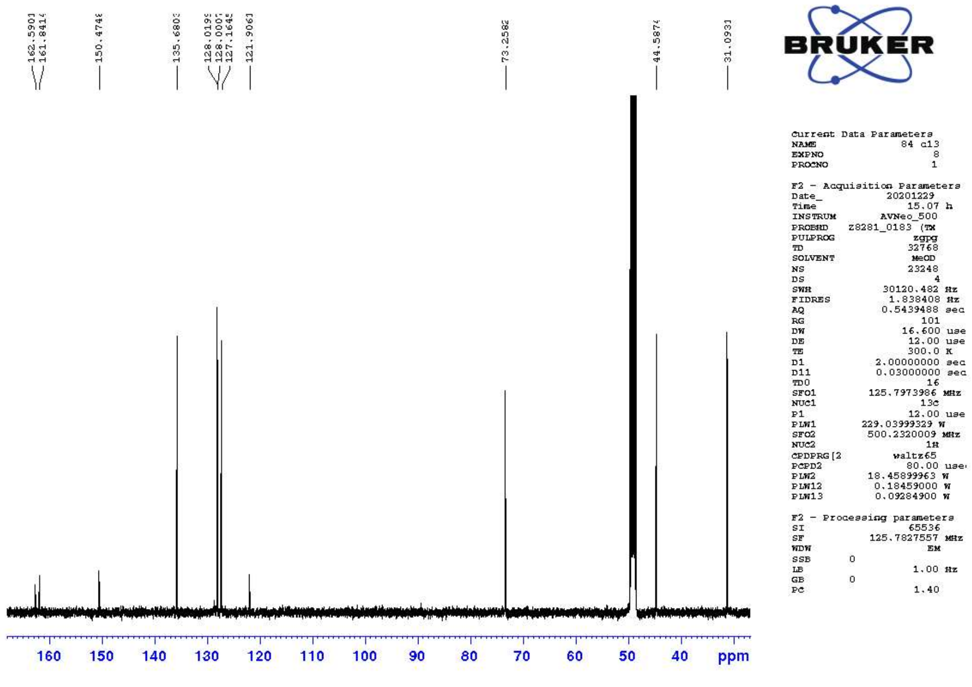

Figure S10: C-13 NMR spectrum of compound **3**.

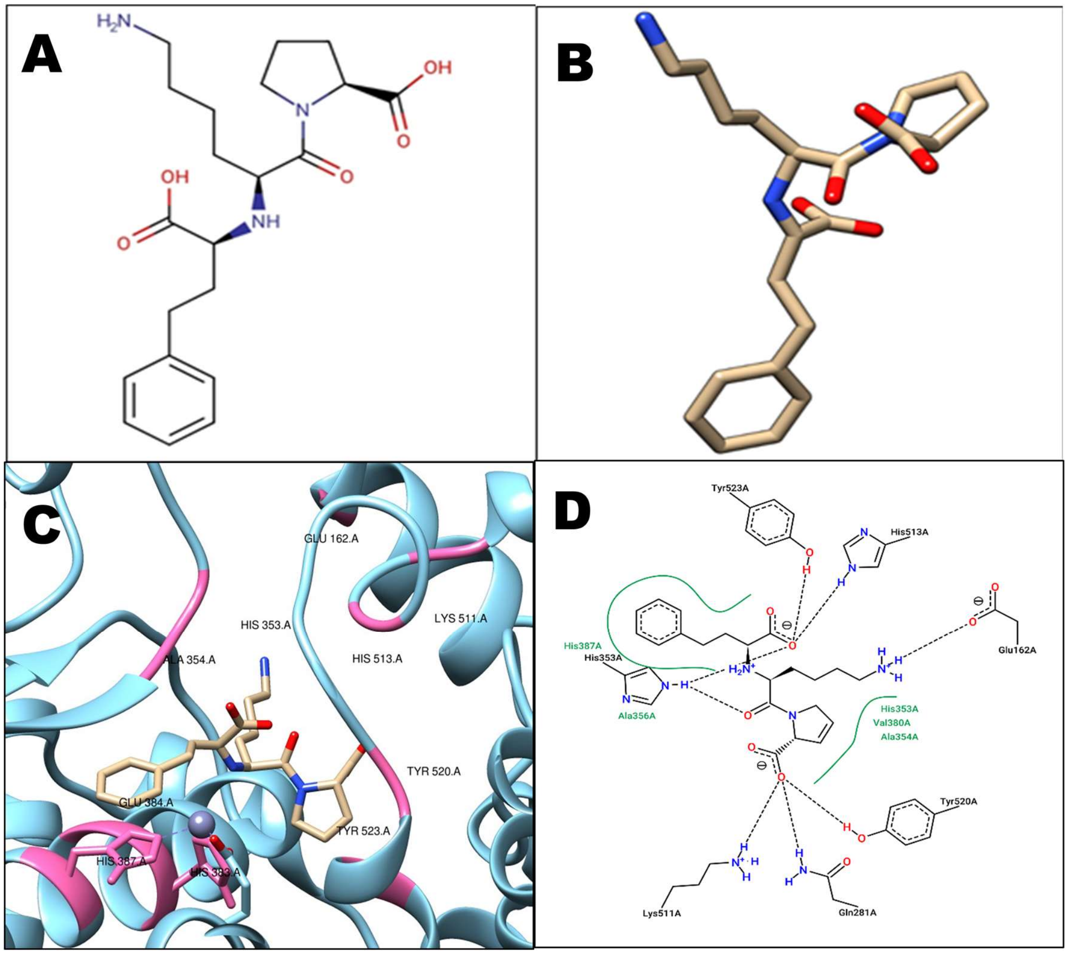

Figure S11: (A) 2D Structure of lisinopril, (B) 3D structure of lisinopril, (C) 3D interaction of lisinopril with ACE generated through Chimera, and (D) 2D interactions of lisinopril with ACE generated through PoseView.

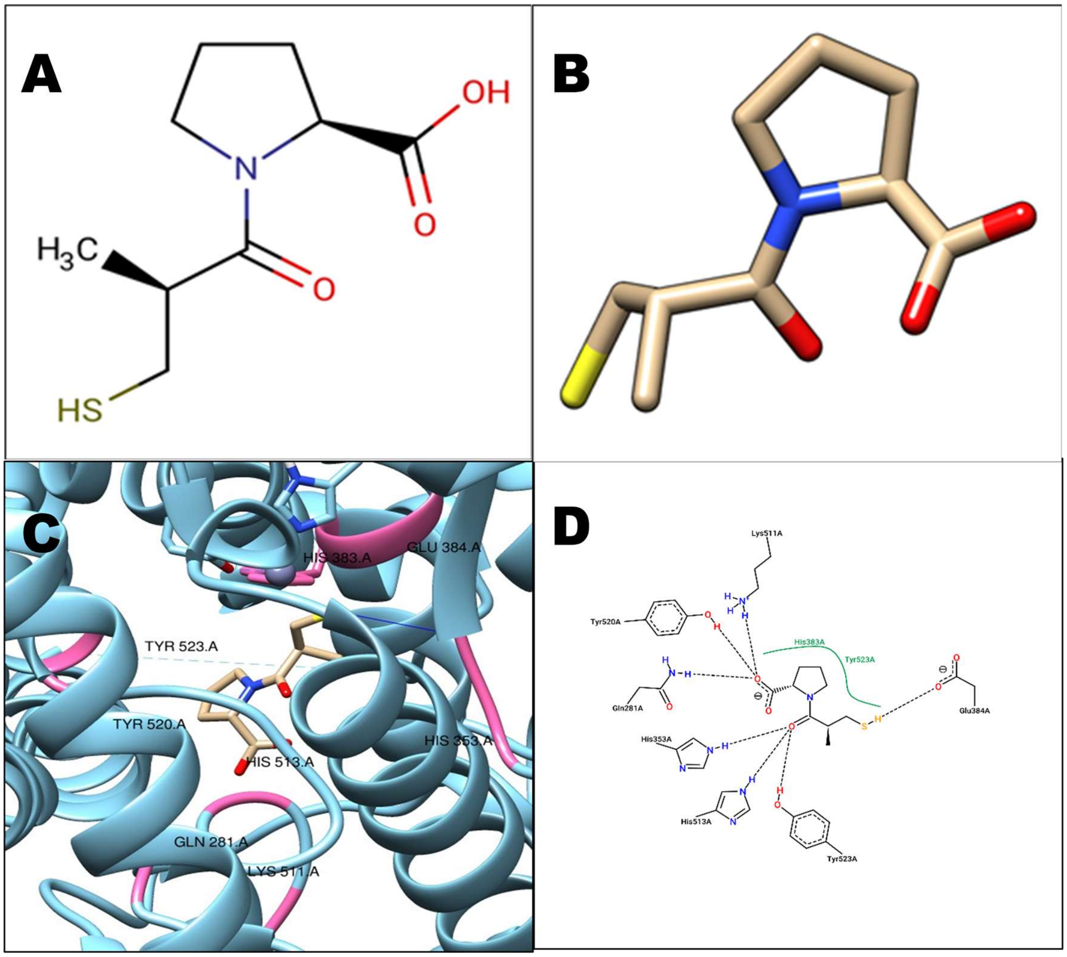

Figure S12: (A) 2D Structure of captopril, (B) 3D structure of captopril (C) 3D interaction of captopril with ACE generated through Chimera, and (D) 2D interactions of captopril with ACE generated through PoseView.

Table S1: Reaction initial velocity of ACE in enzymatic assay.

**Points**	**Cf-Ci (mM)**	**tf-ti (min)**	**v⁰ (mM/min)**
1	0.750	10	0.0750
2	0.371	10	0.0371
Average reaction velocity for initial 20 minutes			0.056

Table S2: ACE inhibitory activity of plant extracts.

**S. No.**	**Code**	**Name of plant**	**%Inhibitory activity**	**Activity**

1	AG	*Alpinia galangal*	6.15	Active
2	ZV	*Ziziphus vulgaris*	25.92	Active
3	BO	*Borago officinalis*	10.08	Active
4	AO	*Althaea officinalis*	6.69	Active
5	CL	*Cordia latifolia*	8.60	Active
6	VO	*Viola odorata*	> 5%	In active
7	GG	*Glycyrrhiza glabra*	> 5%	In active
8	PL	*Piper longum*	> 5%	In active
9	AV	*Adhatoda vasica*	10.15	Active
10	HO	*Hyssopus officinalis*	> 5%	In active
11	AT	*Anemone tetrasepala*	10.52	Active
12	AN	*Anemone obtusiloba*	> 5%	In active
13	AF	*Aquliegia fragrans*	12.82	Active
14	CQ	*Cissus quadrangularis*	9.78	Active
15	FI	*Fagonia indica*	11.45	Active
16	EO	*Emblica officinalis*	> 5%	In active
17	CA	*Centella asciatica*	> 5%	In active
18	AS	*Amomum subulatum*	> 5%	In active
19	DD	*Delphinium denudatum*	> 5%	In active
20	LA	*Lavandula angustifolia*	> 5%	In active
21	CD	*Canscora decussate*	> 5%	In active
22	GB	*Ginko biloba*	> 5%	In active
23	HM	*Herpestis monniera*	> 5%	In active
